# Early detection of myocardial ischemia in resting ECG: analysis by HHT

**DOI:** 10.1186/s12938-023-01089-9

**Published:** 2023-03-10

**Authors:** Chun-Lin Wang, Chiu-Chi Wei, Cheng-Ting Tsai, Ying-Hsiang Lee, Lawrence Yu-Min Liu, Kang-Ying Chen, Yu-Jen Lin, Po-Lin Lin

**Affiliations:** 1 Ph.D. Program of Technology Management, Chung Hua University, Hsinchu, 30012 Taiwan, ROC; 2 Department of Industrial Management, Chung Hua University, Hsinchu, 30012 Taiwan, ROC; 3 Cardiovascular Center, MacKay Memorial Hospital, No. 92, Sec. 2, Zhongshan N. Rd., Zhongshan Dist., Taipei City, 104217 Taiwan, ROC; 4 Department of Cosmetic Applications and Management, MacKay Junior College of Medicine, Nursing, and Management, Taipei City, 112021 Taiwan, ROC; 5 Department of Medicine, MacKay Medical College, No. 46, Sec. 3, Zhongzheng Rd., Sanzhi Dist., New Taipei City, 252005 Taiwan, ROC; 6 Department of Artificial Intelligence and Medical Application, MacKay Junior College of Medicine, Nursing, and Management, No. 46, Sec. 3, Zhongzheng Rd., Sanzhi Dist., New Taipei City, 252005 Taiwan, ROC; 7 Taoyuan Metro Corporation, Taoyuan City, 337601 Taiwan, ROC; 8 Big Light Optics Co., Ltd, Zhubei City, Hsinchu, 302051 Taiwan, ROC; 9 Division of Cardiology, Department of Internal Medicine, Hsinchu MacKay Memorial Hospital, No. 690, Sec.2, Guangfu Rd., East Dist., Hsinchu City, 30071 Taiwan, ROC; 10 Department of Nursing, MacKay Junior College of Medicine, Nursing, and Management, No. 92, Shengjing Rd., Beitou Dist., Taipei City, 112021 Taiwan, ROC

**Keywords:** Exercise electrocardiography, Hilbert–Huang transform, Power spectral density, Myocardial energy

## Abstract

**Background:**

Exercise electrocardiography (ECG) is a noninvasive test aiming at producing ischemic changes. However, resting ECG cannot be adopted in diagnosing myocardial ischemia till ST-segment depressions. Therefore, this study aimed to detect myocardial energy defects in resting ECG using the Hilbert–Huang transformation (HHT) in patients with angina pectoris.

**Methods:**

Electrocardiographic recordings of positive exercise ECG by performing coronary imaging test (n = 26) and negative exercise ECG (n = 47) were collected. Based on the coronary stenoses severity, patients were divided into three categories: normal, < 50%, and ≥ 50%. During the resting phase of the exercise ECG, all 10-s ECG signals are decomposed by HHT. The RT intensity index, composed of the power spectral density of the P, QRS, and T components, is used to estimate the myocardial energy defect.

**Results:**

After analyzing the resting ECG using HHT, the RT intensity index was significantly higher in patients with positive exercise ECG (27.96%) than in those with negative exercise ECG (22.30%) (p < 0.001). In patients with positive exercise ECG, the RT intensity index was gradually increasing with the severity of coronary stenoses: 25.25% (normal, n = 4), 27.14% (stenoses < 50%, n = 14), and 30.75% (stenoses ≥ 50%, n = 8). The RT intensity index of different coronary stenoses was significantly higher in patients with negative exercise ECG, except for the normal coronary imaging test.

**Conclusions:**

Patients with coronary stenoses had a higher RT index at the resting stage of exercise ECG. Resting ECG analyzed using HHT could be a method for the early detection of myocardial ischemia.

**Supplementary Information:**

The online version contains supplementary material available at 10.1186/s12938-023-01089-9.

## Background

Cardiovascular diseases (CVDs), including coronary artery disease (CAD), are the leading cause of death globally with an unmet need on decreasing mortality [1, 2]. CAD is pathologically characterized by atherosclerotic plaque accumulation in the epicardial arteries, whether obstructive or non-obstructive. Electrocardiography (ECG) measures the myocardial electrical activity widely used to detect CAD [3–6]. CAD or myocardial ischemia is usually diagnosed based on the detection of repolarization abnormalities of ECG signals, mainly during ST-segment depressions [7]. Pathological Q waves or left bundle branch block could be another indirect sign of CAD during resting ECG. If a patient has a high clinical likelihood of CAD without significant ischemia changes during resting ECG, an exercise ECG test is suitable for measuring the initial, middle, and post-ECG exercise while strengthening the heart’s oxygen consumption [7]. As the patient’s body works harder during the exercise ECG test, limitation of the coronary blood flow due to obstructive CAD may result in ischemia changes during ECG. However, exercise ECG has limited the power to confirm obstructive CAD [7]. Muscle contraction, baseline wander, and powerline interference will interfere with ECG signals during ECG analysis [8]. If CAD cannot be excluded by clinical assessment, using noninvasive coronary computed tomography is recommended to establish the diagnosis [7, 9].

Additional analysis of ECG signals could be another method to improve the accuracy of the ECG exercise test. Various methods have been used to extract ECG features to diagnose a clinical disease, including Hilbert–Huang transformation (HHT) [10], which applies nonlinear and nonstationary signals and is a relatively new method used in biomedical data analysis [10]. Statistical characteristics are extracted from instinct mode functions obtained by applying HHT to R–R intervals. HHT decomposes the original ECG signals through the empirical mode decomposition (EMD) for myocardial energy analysis [11], and has been extensively used in disease detection, including heart failure, atrial fibrillation and CAD [10]. The addition of HHT in patients with suspected CAD could enhance the accuracy; however, the difference in myocardial ischemia in different stages of the ECG exercise test was less discussed [7, 10, 11]. This study aimed to detect the level of myocardial energy defect in resting ECG and analyze the noninvasive HHT test to predict CAD.

## Results

**Fig. 1:**
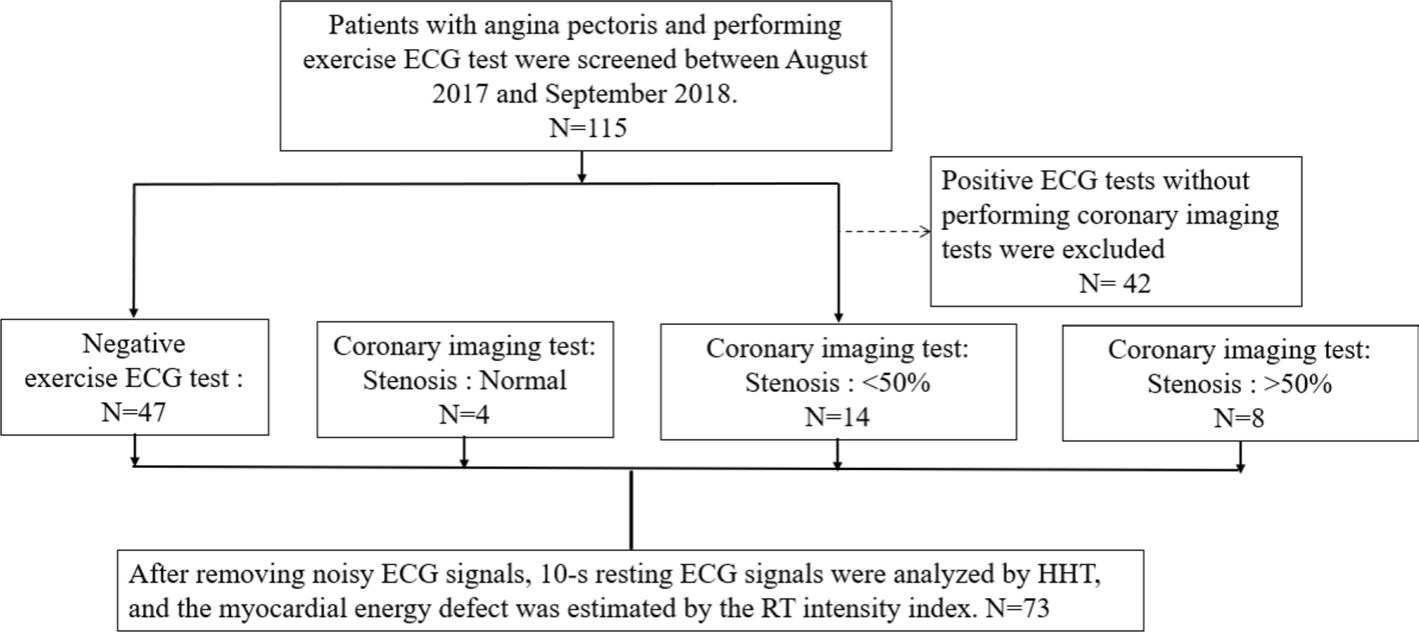
Flow diagram of patient selection

After excluding the positive exercise ECG without coronary imaging test, a total of 73 patients who underwent exercise ECG were enrolled in the study, consisting of 47 patients (male: 34 and female: 13) with negative ECG as a reference group. Based on the coronary stenoses severity, patients with positive ECG exercise (n = 26; male: 16 and female: 10) were divided into three groups: normal (n = 4), < 50% (n = 14), and ≥ 50% (n = 8) stenoses (Fig. 1).

Compared with the reference group of negative exercise ECG, the following analysis models were designed: model 1, positive exercise ECG (n = 26); model 2, stenoses < 50% (n = 18) and ≥ 50% (n = 8) stenoses; and model 3, normal (n = 4), < 50% (n = 14), and ≥ 50% (n = 8) stenoses. All resting-stage recordings of exercise ECG were analyzed using HHT, and the myocardial energy defect was estimated using the RT intensity index (Table 1, Additional files 3, 5, 6, 7, 8, 11, 12 and 13).

**Table 1:** The severity distribution of patients between groups

Exercise ECG tests	Number	RT Index (%)	Std. error (%)	Upper bound (%)	Lower bound (%)
Negative	47	22.30	0.63	23.57	21.02
Positive					
Stenoses: 0%	4	25.25	2.95	34.65	15.85
Stenoses: < 50%	14	27.14	1.40	30.16	24.12
Stenoses: ≥ 50%	8	30.75	2.60	36.91	24.59

**Fig. 2:**
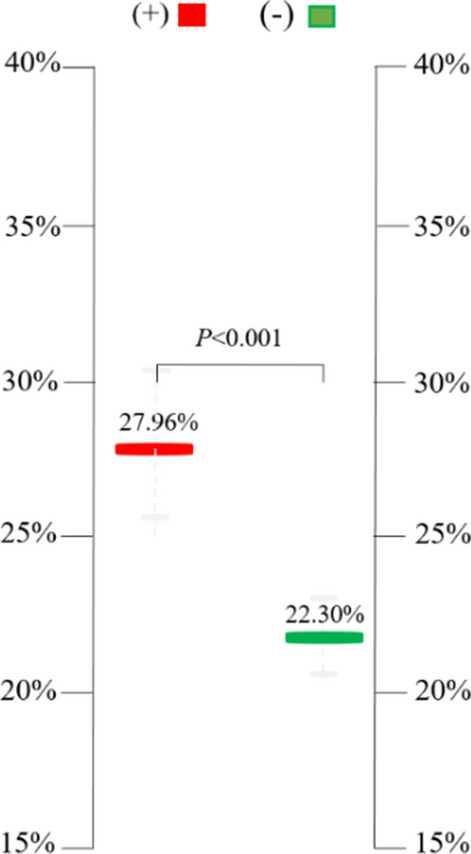
The difference in myocardial electrical energy (RT index) between the positive and negative exercise ECG groups during the resting stage (Model 1)

**Model 1**: Comparing the energy difference of resting exercise ECG energy between the positive and negative exercise groups, the RT intensity index of the positive exercise ECG group (27.96% ± 6.14%) was significantly higher than that of the negative group (22.30% ± 4.34%) (p < 0.001) (Fig. 2, Table 2 and Additional file 1).

**Table 2:** The difference in RT index between negative and positive exercise ECG groups

Variable	Exercise ECG	Number	Male	Female	Age	RT index (%)	Std. deviation (%)	Std. error (%)	Upper bound (%)	Lower bound (%)	p-value
Threshold	Negative	47	34	13	50.68	22.30	4.34	0.63	23.57	21.02	
Model 1	Positive	26	16	10	49.38	27.96	6.14	1.20	30.44	25.48	< 0.001

**Fig. 3:**
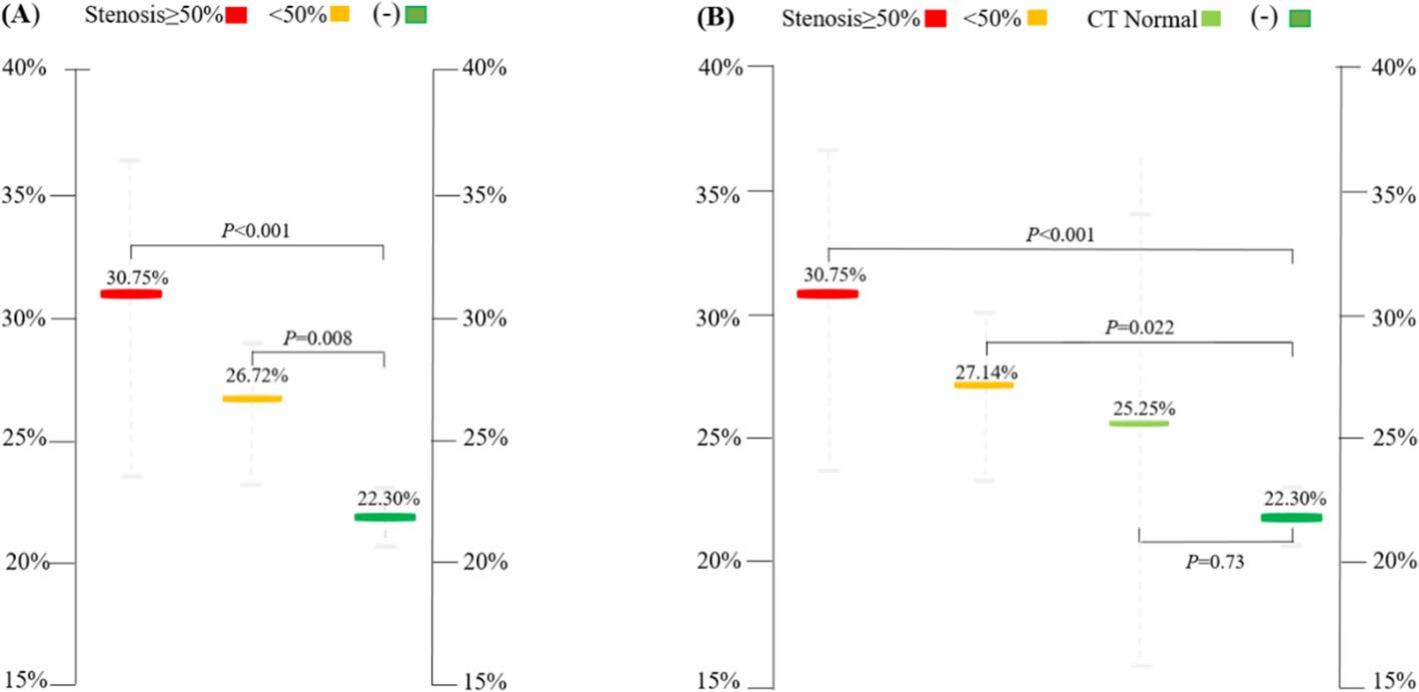
The difference in myocardial electrical energy (RT index) between negative and positive exercise ECG groups with different coronary stenoses in the resting stage (**A**: model 2 and **B**: model 3, Additional files 4, 9, 10)

**Model 2**: Based on the coronary stenoses severity, patients with positive exercise ECG (n = 26) were divided into two groups: < 50% (N = 18) and ≥ 50% (N = 8) coronary stenoses (Fig. 3-A). The RT index of the group with < 50% (26.72% ± 5.27%) or ≥ 50% (30.75% ± 7.36%) coronary stenoses was significantly higher than that in the negative group (22.30% ± 4.34%) (p = 0.008 and p < 0.001) (Table 3, Additional file 2).

**Table 3:** The difference in RT index based on different degrees of coronary stenoses

Variable	Exercise ECG	Number	Male	Female	Age	RT index (%)	Std. deviation (%)	Std. error (%)	Upper bound (%)	Lower bound (%)	p-value
Threshold	Negative	47	34	13	50.68	22.30	4.34	0.63	23.57	21.02	
Model 1	Positive	26	16	10	49.38	27.96	6.14	1.20	30.44	25.48	< 0.001
	Coronary stenoses										
Model 2	< 50%	18	11	7	47.6	26.72	5.27	1.24	29.34	24.10	0.008
	≥ 50%	8	5	3	53.38	30.75	7.36	2.60	36.91	24.59	< 0.001
Model 3	Normal	4	3	1	42.75	25.25	5.91	2.95	34.65	15.85	0.73
	< 50%	14	8	6	49	27.14	5.23	1.40	30.16	24.12	0.022
	≥ 50%	8	5	3	53.38	30.75	7.36	2.60	36.91	24.59	< 0.001

**Fig. 4:**
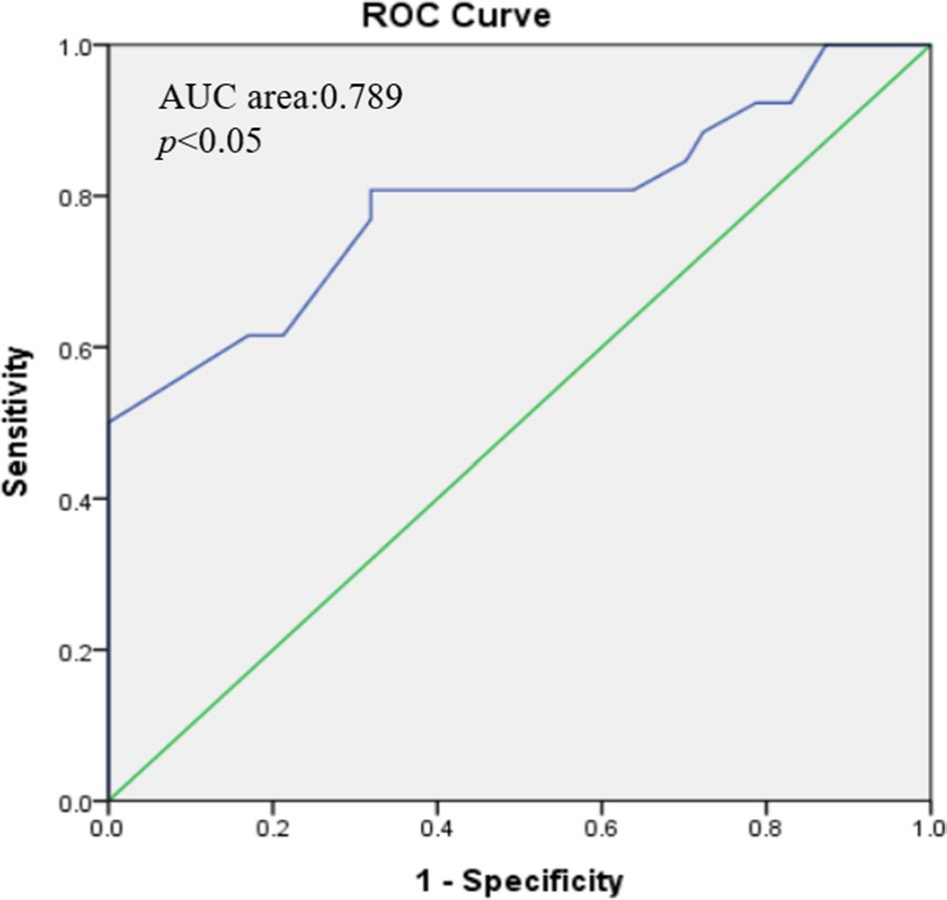
Analysis of the receiver-operating (AUC) characteristics curve for differentiating between myocardial ischemia and different coronary stenoses using the RT index during the resting stage of the exercise ECG

**Model 3**: Based on coronary stenoses severity, patients with positive exercise ECG (n = 26) were divided into three groups: normal (n = 4), < 50% (n = 14), and ≥ 50% (n = 8) stenoses < 50% (Fig. 3-B). No significant difference in the RT index was observed between the negative exercise ECG (22.30% ± 4.34%) and normal coronary image groups (25.25% ± 5.91%; p = 0.73). The RT index of coronary stenoses of < 50% (27.14% ± 5.23%) or ≥ 50% (30.75% ± 7.36%) was significantly higher (p = 0.022 and p < 0.001) (Table 3). The diagnostic accuracy of the RT index during the resting phase of the exercise ECG for differentiating between myocardial ischemia and different coronary stenoses, as quantified by the area under the receiver-operating characteristics curve (AUC) was 0.789 (p < 0.05) (Fig. 4).

## Discussion

The main findings of the present study indicated that patients with significant coronary stenoses had a higher RT index in the resting stage of exercise ECG. Moreover, resting ECG signals analyzed by HHT could be a method of the early detection of myocardial energy defects in patients with angina pectoris.

According to the 2019 European Society of Cardiology guidelines for the diagnosis and management of chronic coronary syndromes, a resting ECG is useful for the early detection of CAD [7, 12]. A resting ECG is an important tool for diagnosing myocardial ischemia, whereas dynamic ST-segment changes are recorded during ongoing angina or myocardial infarction. Although even ambulatory ECG monitoring is capable of detecting silent ischemia in chronic coronary syndrome patients, resting ECG cannot replace exercise ECG as it can rarely provide any information that could help in the diagnosis or prognosis of myocardial ischemia [7]. Therefore, an exercise ECG with increased myocardial oxygen consumption may be considered in patients with a clinical likelihood of CAD. However, exercise ECG has very limited diagnostic power in detecting obstructive CAD, compared with coronary imaging tests [7, 13]. Besides, a resting or exercise ECG with pre-existing repolarization abnormalities, such as pathological Q waves or conduction abnormalities, will influence the accuracy of detecting CAD. In an extensive study of 3094 patients for > 12 years (1969–1981), five elements (maximum exercise rhythm, number of exercises, CAD, angina pectoris type, age, and gender) had significant independent effects on exercise ECG testing sensitivity [14]. Exercise ECG combined with the functional image test could confirm the CAD diagnosis; however, the process is more time-consuming or costly [7].

The ECG reflects changes in the myocardial bioelectricity in the R^3^ space in each cardiac cycle and presents the results in the R^2^ space ECG. Muscle contraction, baseline wander, and powerline interference will interfere with ECG signals during analysis [8]. Various methods have been used to extract ECG feature disorders to diagnose a clinical disease, including wavelet analysis, discrete Fourier transform, EMD, second-order difference plot, wavelet packet decomposition, or HHT [2, 10, 12, 15–25]. The EMD has a large frequency distribution and high sample rate, especially in long time series [26], and is computationally expensive with some serious disadvantages such as border effects and mode mixing [27–30]. WT/FFT employs an integral method on a pre-established basis via the inner product method to determine the content of the base component in the data as a representative of the spectrum intensity, and the change of the disturbance frequency or wavelength may have occurred prior to the entire period of disturbance. The occurrence (the so-called intra-wave). HHT uses a differential method to decompose the signal into several IMFs with good symmetry properties. The perturbation frequency and amplitude of each IMF can change with time, and the manner of change is completely determined by the characteristics of the data itself. This basis is not pre-settled. Which is fixed and obtained by decomposing the signal itself through EMD, which belongs to the post-basis. The instantaneous frequency obtained by HHT has a clear physical meaning and can characterize the signal’s local characteristics [31]. Despite the limited theoretical analysis, nonlinear and nonstationary signal problems can be solved with HHT [4, 5, 32, 33]. HHT with EMD and Hilbert spectral analysis has been extensively used to analyze nonstationary signals, including heart failure, atrial fibrillation, and CAD [10]. Feature extraction of ECG signals using the HHT algorithm was independent of the 12-lead ECG detection method and had been widely used for detecting the myocardial energy defect [34, 35]. Patients with CAD could be distinguished from normal patients using features obtained by applying wavelet transformation and power spectral destiny for R–R intervals [10]. When comparing C1, C2, and C3 decomposed from pulse wave, no significant difference was observed in component C1 between healthy adults and patients with CAD but showed significant differences in component C3 (p < 0.05) [6]. Using the HHT algorithm in exercise ECG could improve the accuracy of diagnosis of myocardial ischemia [11]

In our previous study, we used two methods:

Method 1: Different stages exercise ECG before/during/post.
Method 2: Only use resting ECG before exercise.

The study reported that applying the HHT algorithm with the RT intensity and ST kinetic energy indices could reduce the pseudo-positive rate by 83.3%, as compared with exercise ECG only [11]. The ST kinetic energy index contained C4 and C5 components and is more sensitive to the QRS energy changes. Therefore, the ST kinetic energy index reflected a significant energy defect during the exercise stage of ECG [11]. However, a significant difference in the RT intensity index in the resting stage of exercise ECG was observed between patients with negative and positive exercise ECG [11]. In our study, we also found the myocardial energy defect in the resting stage of exercise ECG via the RT intensity index. In patients with positive exercise ECG, the RT intensity index was gradually increasing with the coronary stenoses severity. No significant difference in the RT intensity index was observed between positive exercise ECG with normal coronary imaging test and negative exercise ECG.

To the best of our knowledge, this was the first study to evaluate the relationship between coronary stenoses and myocardial energy difference of HHT in the resting stage of exercise ECG. Several study limitations should be considered in this study, i.e., the absence of prospective power and the small sample sizes. Therefore, we could not assess the benefits of detecting the early energy defect in the high-risk group based on future events. Further studies may investigate the accuracy, sensitivity, and specificity of the RT intensity index in predicting CAD during a resting ECG, as well as the effect of the differences between different ages and genders.

## Conclusions

The resting stage of exercise ECG signal combined with HHT analysis can predict myocardial ischemia before the exercise stage. Patients with severe coronary stenoses had a higher RT index during the resting stage of exercise ECG than those with negative exercise ECG and normal or insignificant coronary stenoses. The resting ECG analyzed by HHT could be used as a new method for the early detection of myocardial ischemia before exercise ECG tests or invasive imaging studies.

## Methods

### Study population

In this retrospective study, all patients with angina pectoris and who underwent exercise ECG tests were screened between August 2017 and September 2018. The Institutional Review Board of Mackay Memorial Hospital approved this study protocol (IRB No. 17MMHIS004e), which waived the requirement for informed consent in this retrospective study. The treating physicians decided on the need to perform exercise ECG tests after excluding ECG abnormalities, including LBBB, paced rhythm, Wolff–Parkinson–White syndrome, ≥ 0.1-mV ST-segment depression on resting ECG, or who are being treated with digitalis. The use of exercise ECG test was indicated by treating physicians and re-confirmed by other two cardiologists. Patients with positive exercise ECG were suggested to undergo coronary imaging, including coronary angiography or computed tomography. Based on the coronary stenoses severity, patients with positive exercise ECG were divided into three groups: normal, < 50%, and ≥ 50% stenoses. According to 2019 ESC Guidelines for the diagnosis and management of chronic coronary syndromes [7], the negative predictive value of exercise ECG was higher than positive predictive value. The likelihood of CAD was less than 15% if negative exercise ECG. Therefore, patients with negative exercise ECG were defined as a relative health group. Compared with patients with negative exercise ECG, analysis models were designed (model 1, positive exercise ECG; model 2, < 50% and ≥ 50% stenoses; and model 3, normal, < 50%, and ≥ 50% stenoses).

### Resting ECG signal data

**Fig. 5:**
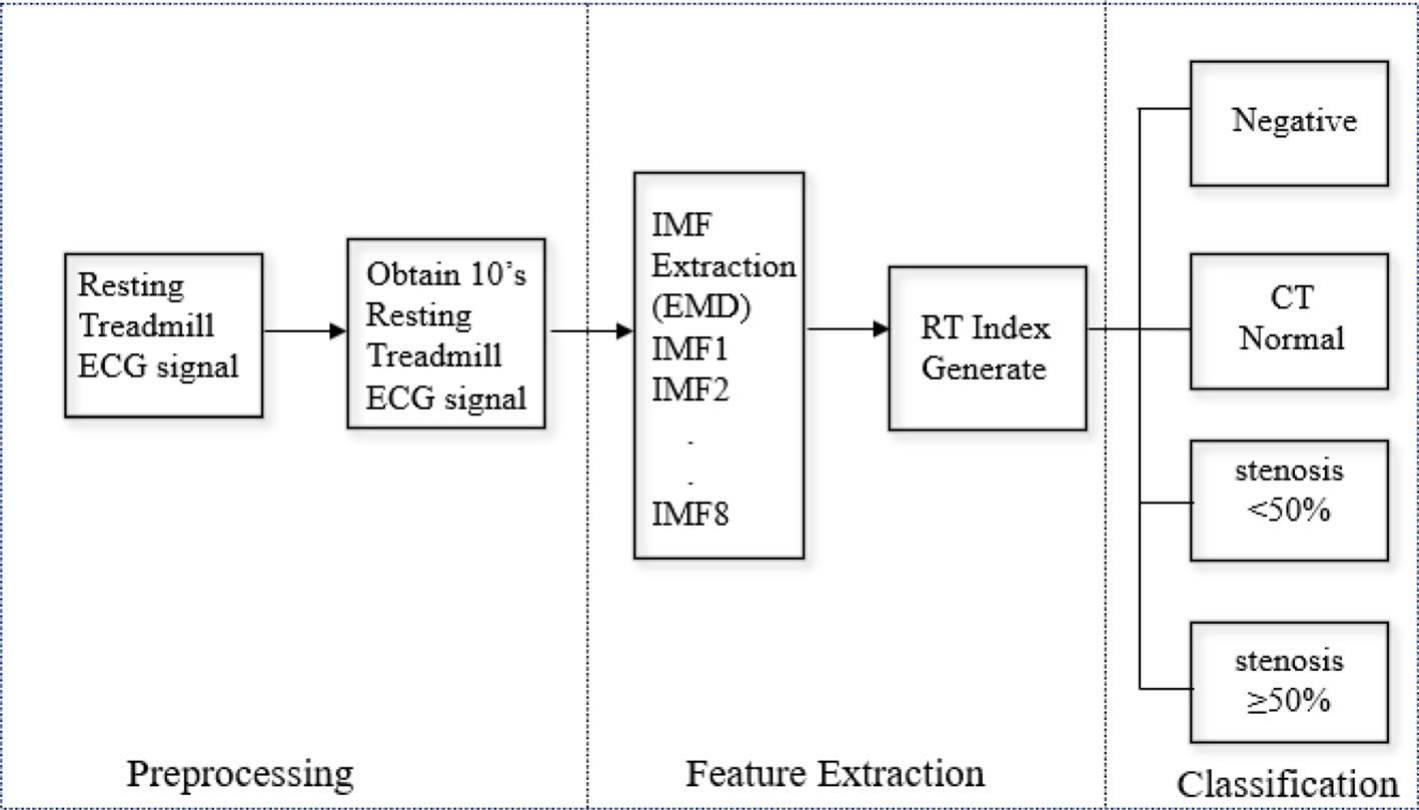
Structure of proposed diagnostic blocks

After removing the baseline drift and the EMG signals in the first 7 s, stable resting ECG signals of 10 s were analyzed by pre-processing, feature extraction then classified by different coronary stenoses severities (Fig. 5).

**Fig. 6:**
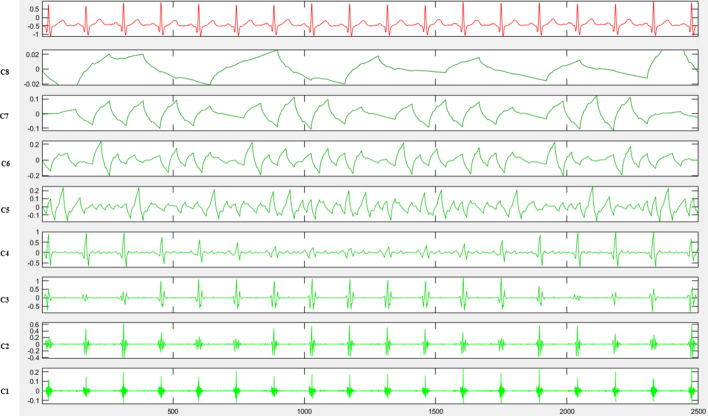
A stable resting ECG signal with a sampling rate of 250 HZ in 10-s cycles is obtained in patients with negative exercise ECG

The main ECG energy is concentrated at 0–40 Hz. The ECG signal can be divided into three components: P, QRS, and T based on waveform characteristics. In this study, the total ECG energy was decomposed into C1–8 spectrum components through EMD (Fig. 6):

- C1 and C2 are the highest frequency decomposition components of the QRS wave.
- C3 adds the P wave’s decomposition components.
- C4 adds the decomposition component of the T wave, resulting from the superposition of three components: P, QRS, and T wave.
- C5 is a superposition of low-frequency components of P, QRS, and T waves.
- C6 represents the cardiac cycle, showing the heartbeat rhythm.
- C7 and C8 are cardiac physiological adjustment rhythms on a long-term scale, representing the heart’s long-term rhythm.

C1, C2, … to Cn represent disturbances from small to large wavelength scales. We can imagine the superposition of these disturbances as a small wave riding on an enormous wave, with still smaller waves on top of each of these small waves. The wavelength, frequency, and amplitude of each wave will change with time. Unlike the base assumed by FFT, the frequency and amplitude are unchanged and constant.

The RT intensity index was defined as:

$${\text{RT}} = \frac{\alpha C1 + \beta C2 + \gamma C3}{{\sum_{i = 1}^8 {Ci} }}$$

α∣C1∣ + β∣C2∣ + γ∣C3∣)/∣C total∣α/β/γ are weighting parameters of empirical correlation following rules

**Fig. 7:**
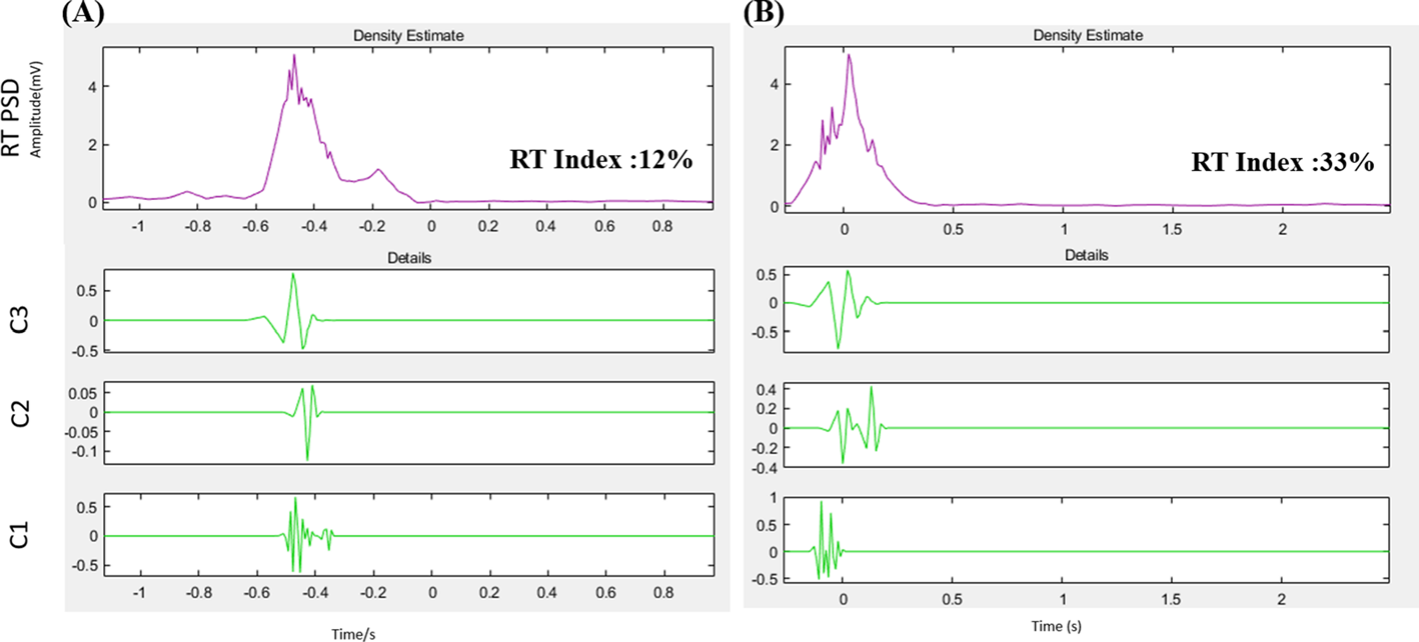
The 10-s resting ECG signals were analyzed by the RT intensity index in patients with **A** negative exercise ECG and with **B** positive exercise ECG with coronary stenoses

1. (α + β + γ) is less than 1.
2. α ≥ β ≥ γ.

the R wave intensity ratio (C1, C2, and C3 components compared to the total of C1–8) [11]. The RT index can display myocardial energy changes during resting ECG and is more sensitive in the QRS complex. All resting-stage electrocardiographic recordings of exercise ECG were analyzed using the RT intensity index (Fig. 7).

### Statistical analysis

A two-way analysis of variance for repeated measures followed by a Bonferroni post-hoc test was used to analyze the RT index of resting-stage exercise ECG. The RT intensity index of negative exercise ECG was defined as a negative threshold. RT intensity indices of patients with positive exercise ECG were divided into three models based on the coronary stenoses severity. Compared with the negative threshold of the RT index, these models were used to analyze the positive exercise ECG group. Statistical significance was set at p < 0.05. All data analyses were performed using IBM SPSS statistics 20 for windows (IBM Corp., Chicago, IL, USA).

## Supplementary Information

**Additional file 1.** CT ≥ 50% * 8.docx

**Additional file 2.** CT ≥ 50% * 8.xlsx

**Additional file 3.** CT < 50% *14.docx

**Additional file 4.** CT < 50% *14.xlsx

**Additional file 5.** CT Normal * 4.docx

**Additional file 6.** CT Normal * 4.xlsx

**Additional file 7.** model 1 RT ANOVA.spv

**Additional file 8.** model 2 RT ANOVA.spv

**Additional file 9.** model 3 RT ANOVA.spv

**Additional file 10.** Negative * 47.docx

**Additional file 11.** Negative * 47.xlsx

**Additional file 12.** RT ANOVA.sav

**Additional file 13.** RT Intensity Index.xlsx

## Data Availability

Datasets used and/or analyzed during the current study are available from the corresponding author upon reasonable request.

## Abbreviations

CAD: Coronary artery disease
CVDs: Cardiovascular disease
ECG: Electrocardiography
EMD: Empirical mode decomposition
HHT: Hilbert–Huang transformation
