# Supplementary material for: Early detection of myocardial ischemia in resting ECG: analysis by HHT
Source: Biomed Eng Online. 2023 Mar 10;22:23. doi: 10.1186/s12938-023-01089-9 (PMC9999640; doi:10.1186/s12938-023-01089-9)
Supplement: Supplementary file 1 — Additional file 1. CT ≥ 50% * 8.docx [file 12938_2023_1089_MOESM1_ESM.docx]

CT ≥50% * 8

| Number | Gender | Age | Medical Order | RT intensity index |
| --- | --- | --- | --- | --- |
| CT≥50%001 | 1 | 44 | SVD、d-RCA55%、Hyperlipidemia、Diabetes | 36% |
| CT≥50%002 | 2 | 61 | CTA: P-LAD5=50-69%、Hypertension | 29% |
| CT≥50%003 | 2 | 54 | p-LAD 50% stenosis、Hyperlipidemia | 30% |
| CT≥50%004 | 1 | 43 | TVD、Hypertension、Hyperlipidemia | 21% |
| CT≥50%005 | 1 | 46 | LAD(Ostium) 80% stenosis、Hypertension、Hyperlipidemia | 33% |
| CT≥50%006 | 1 | 59 | DVD、d-LCX、75%、Hyperlipidemia、Diabetes | 45% |
| CT≥50%007 | 1 | 59 | Hyperlipidemia、Diabetes、2015  CTA + Angiography (VGH) : CAD, DVD (p-RCA 70%, m-LAD 50%) | 26% |
| CT≥50%008 | 2 | 61 | m-LAD 75% stenosis post PCI + stent (Biofreedom 2.75x 24) | 26% |
