# Supplementary material for: Early detection of myocardial ischemia in resting ECG: analysis by HHT
Source: Biomed Eng Online. 2023 Mar 10;22:23. doi: 10.1186/s12938-023-01089-9 (PMC9999640; doi:10.1186/s12938-023-01089-9)
Supplement: Supplementary file 3 — Additional file 3. CT < 50% *14.docx [file 12938_2023_1089_MOESM3_ESM.docx]

CT<50% *14

| Number | Gender | Age | Medical Order | RT intensity index |
| --- | --- | --- | --- | --- |
| CT<50%001 | 1 | 57 | p-RCA 30% stenosis、Hyperlipidemia、Hypertension | 26% |
| CT<50%002 | 1 | 57 | CTA: Myocardial bridge、Hyperlipidemia、Hypertension | 28% |
| CT<50%003 | 1 | 43 | CTA: P-LAD 25-50%、Hyperlipidemia、Hypertension | 28% |
| CT<50%004 | 1 | 48 | p-LAD 30% and p-LCX 30% stenosis、Hyperlipidemia、Hypertension、HX | 30% |
| CT<50%005 | 1 | 38 | m-LAD 30% stenosis , m-LAD bridge formation、Hyperlipidemia、Hypertension、Diabetes | 33% |
| CT<50%006 | 2 | 41 | Hyperlipidemia、A short segmental myocardial bridge at the distal third of LAD | 17% |
| CT<50%007 | 2 | 45 | m-LAD 20% stenosis、Hyperlipidemia | 19% |
| CT<50%008 | 1 | 53 | LCX-P : 28 %、Hyperlipidemia、Hypertension | 20% |
| CT<50%009 | 2 | 55 | Hyperlipidemia、Patent coronary artery TIMI 2 flow of LAD、TIMI | 26% |
| CT<50%010 | 2 | 48 | p-LAD 30% stenosis、Hyperlipidemia、Hypertension、Diabetes | 30% |
| CT<50%011 | 1 | 54 | CTA: LAD bridge、Hyperlipidemia、Hypertension | 31% |
| CT<50%012 | 2 | 44 | d-LAD 30% stenosis、Hyperlipidemia、Hypertension | 35% |
| CT<50%013 | 2 | 50 | Patent coronary artery : slow flow、Hyperlipidemia | 29% |
| CT<50%014 | 1 | 53 | Hyperlipidemia、Hypertension、Patent coronary artery : slow flow | 28% |
