# Supplementary material for: Early detection of myocardial ischemia in resting ECG: analysis by HHT
Source: Biomed Eng Online. 2023 Mar 10;22:23. doi: 10.1186/s12938-023-01089-9 (PMC9999640; doi:10.1186/s12938-023-01089-9)
Supplement: Supplementary file 5 — Additional file 5. CT Normal * 4.docx [file 12938_2023_1089_MOESM5_ESM.docx]

CT Normal * 4

| Number | Gender | Age | Medical Order | RT intensity index |
| --- | --- | --- | --- | --- |
| CT Normal 001 | 2 | 37 | CTA: normal | 30% |
| CT Normal 002 | 1 | 52 | CTA: normal、Hyperlipidemia | 17% |
| CT Normal 003 | 1 | 38 | CTA normal、Hyperlipidemia 、 Hypertension | 25% |
| CT Normal 004 | 1 | 44 | CTA: normal、Hyperlipidemia | 29% |
