# Supplementary material for: Early detection of myocardial ischemia in resting ECG: analysis by HHT
Source: Biomed Eng Online. 2023 Mar 10;22:23. doi: 10.1186/s12938-023-01089-9 (PMC9999640; doi:10.1186/s12938-023-01089-9)
Supplement: Supplementary file 10 — Additional file 10. Negative * 47.docx [file 12938_2023_1089_MOESM10_ESM.docx]

Negative * 47

| Number | Gender | Age | Medical Order | RT intensity index |
| --- | --- | --- | --- | --- |
| Negative001 | 1 | 39 | Hyperlipidemia | 12% |
| Negative002 | 2 | 39 |  | 15% |
| Negative003 | 1 | 48 | Hyperlipidemia、 Hypertension | 15% |
| Negative004 | 1 | 43 | Hyperlipidemia | 15% |
| Negative005 | 2 | 58 | Hyperlipidemia、 Hypertension | 16% |
| Negative006 | 1 | 42 |  | 16% |
| Negative007 | 1 | 53 | Hyperlipidemia、 Hypertension | 17% |
| Negative008 | 1 | 40 |  | 17% |
| Negative009 | 1 | 76 | 103.7 Wei-Go CAD, SVD、 Hyperlipidemia、 Hypertension | 18% |
| Negative010 | 2 | 48 | Hyperlipidemia、 Hypertension | 18% |
| Negative011 | 1 | 44 | Hypertension | 19% |
| Negative012 | 1 | 78 |  | 19% |
| Negative013 | 1 | 49 | Hyperlipidemia、 Hypertension | 19% |
| Negative014 | 1 | 49 | Hyperlipidemia、 Hypertension | 20% |
| Negative015 | 1 | 44 | Hyperlipidemia、 Hypertension | 21% |
| Negative016 | 1 | 32 | Hyperlipidemia | 21% |
| Negative017 | 2 | 77 |  | 21% |
| Negative018 | 1 | 42 | Hyperlipidemia | 22% |
| Negative019 | 2 | 47 |  | 22% |
| Negative020 | 2 | 53 |  | 22% |
| Negative021 | 1 | 32 |  | 22% |
| Negative022 | 1 | 20 |  | 22% |
| Negative023 | 1 | 59 | Hyperlipidemia、 Hypertension | 22% |
| Negative024 | 1 | 56 | Diabetes、 Hypertension、Hyperlipidemia | 22% |
| Negative025 | 1 | 56 | Hyperlipidemia | 23% |
| Negative026 | 2 | 61 | Diabetes、 Hypertension、Hyperlipidemia | 23% |
| Negative027 | 1 | 57 | Hypertension | 23% |
| Negative028 | 1 | 45 | Diabetes、 Hypertension、Hyperlipidemia | 23% |
| Negative029 | 1 | 58 | Hyperlipidemia | 23% |
| Negative030 | 1 | 63 | Hypertension | 24% |
| Negative031 | 2 | 48 | Hyperlipidemia | 24% |
| Negative032 | 1 | 43 | Hyperlipidemia | 24% |
| Negative033 | 2 | 40 | Diabetes | 26% |
| Negative034 | 1 | 24 |  | 26% |
| Negative035 | 1 | 69 | Hyperlipidemia、 Hypertension | 26% |
| Negative036 | 1 | 48 |  | 26% |
| Negative037 | 2 | 60 | Hyperlipidemia | 26% |
| Negative038 | 1 | 58 | Hyperlipidemia | 27% |
| Negative039 | 2 | 62 |  | 27% |
| Negative040 | 2 | 64 | Hx | 28% |
| Negative041 | 1 | 58 | Hyperlipidemia、 Hypertension | 28% |
| Negative042 | 1 | 58 | Hyperlipidemia | 28% |
| Negative043 | 2 | 50 | Hyperlipidemia、 Hypertension | 28% |
| Negative044 | 1 | 23 |  | 28% |
| Negative045 | 1 | 59 | Diabetes | 28% |
| Negative046 | 1 | 40 |  | 28% |
| Negative047 | 1 | 70 | Hyperlipidemia | 28% |
